# Cyclosporine is a potential curative treatment option for advanced thymoma

**DOI:** 10.1186/s40164-017-0073-6

**Published:** 2017-05-03

**Authors:** Yusuke Isshiki, Hiroaki Tanaka, Yoshio Suzuki, Yukihiro Yoshida

**Affiliations:** 1 Department of Hematology, Chiba University Hospital, 1-8-1 Inohana, Chuo-ku, Chiba, 260-8677 Japan; 2 Department of Hematology, Asahi General Hospital, Chiba, Japan; 3 Department of Clinical Pathology, Asahi General Hospital, Chiba, Japan; 4 Department of Respiratory Surgery, Asahi General Hospital, Chiba, Japan

**Keywords:** Advanced thymoma, Cyclosporine, Pure red cell aplasia

## Abstract

**Background:**

Thymectomy can effectively cure most thymoma patients; however, patients with advanced thymoma typically require chemotherapy, which is associated with limited efficacy in this context. Here we provide the first report of a patient with recurrent thymoma who achieved complete remission (CR) using cyclosporine therapy.

**Case presentation:**

A 63-year-old woman who had undergone resection surgery for recurrent type B1 thymoma developed pure red cell aplasia (PRCA), and CT findings revealed thymoma recurrence. After the initiation of orally-administered cyclosporine, PRCA quickly resolved, and the thymoma disappeared without the administration of any anti-thymoma therapy. The patient has remained in CR for over 3 years using only cyclosporine.

**Conclusions:**

This is the first report describing the curative potential of cyclosporine for the treatment of advanced thymoma. Although the mechanism underlying this effect remains unclear, cyclosporine can become a less toxic and more cost-effective treatment option for thymoma compared with conventional therapy. Clinical trials are needed to confirm the therapeutic potential of cyclosporine as a new treatment option for thymoma.

## Background

Thymoma is a common primary tumor of the anterior mediastinum [1]. Standard treatment for thymoma is thymectomy, and the survival rate associated with complete resection in patients with early stage disease is favorable [2, 3]. However, some patients experience disease recurrence due to local invasion and metastases [4]. Standard treatment for advanced or recurrent thymoma is chemotherapy, particularly in patients with unresectable thymoma. Several combinations of cytotoxic drugs have been used in this context, and several of these regimens provide favorable response rates and prolonged survival [5]. However, the efficacy of these regimens is limited by the duration of the response, and they are typically unable to cure patients with advanced thymoma [5].

Pure red cell aplasia (PRCA) or myasthenia gravis (MG) are well-established complications observed in thymoma patients [6], and several studies have demonstrated the efficacy of immunosuppressive therapy for these complications. Cyclosporine is generally used for the treatment of thymoma-associated PRCA with remarkable efficacy [7]. Immunosuppressive therapy is also widely used for the treatment of thymoma-associated MG, particularly for patients with unsatisfactory responses to thymectomy and acetylcholinesterase inhibitors [8]. However, the efficacy of immunosuppressive therapies on the underlying thymoma remains unclear.

Herein, we report a patient with recurrent thymoma and PRCA who achieved complete remission (CR) using only cyclosporine. This is the first report providing evidence of the curative potential of cyclosporine in the treatment of advanced thymoma.

## Case presentation

A 50 year-old woman underwent thymectomy for a 40-mm localized anterior mediastinal tumor diagnosed using chest radiography. The histopathological diagnosis was of a microinvasive thymoma, WHO histologic classification type B1, Masaoka stage II. She had no symptoms of MG, but her serum levels of anti-acetylcholine receptor were elevated (7.5 nmol/l). The patients did not receive adjuvant therapy following the thymectomy.

**Fig. 1:**
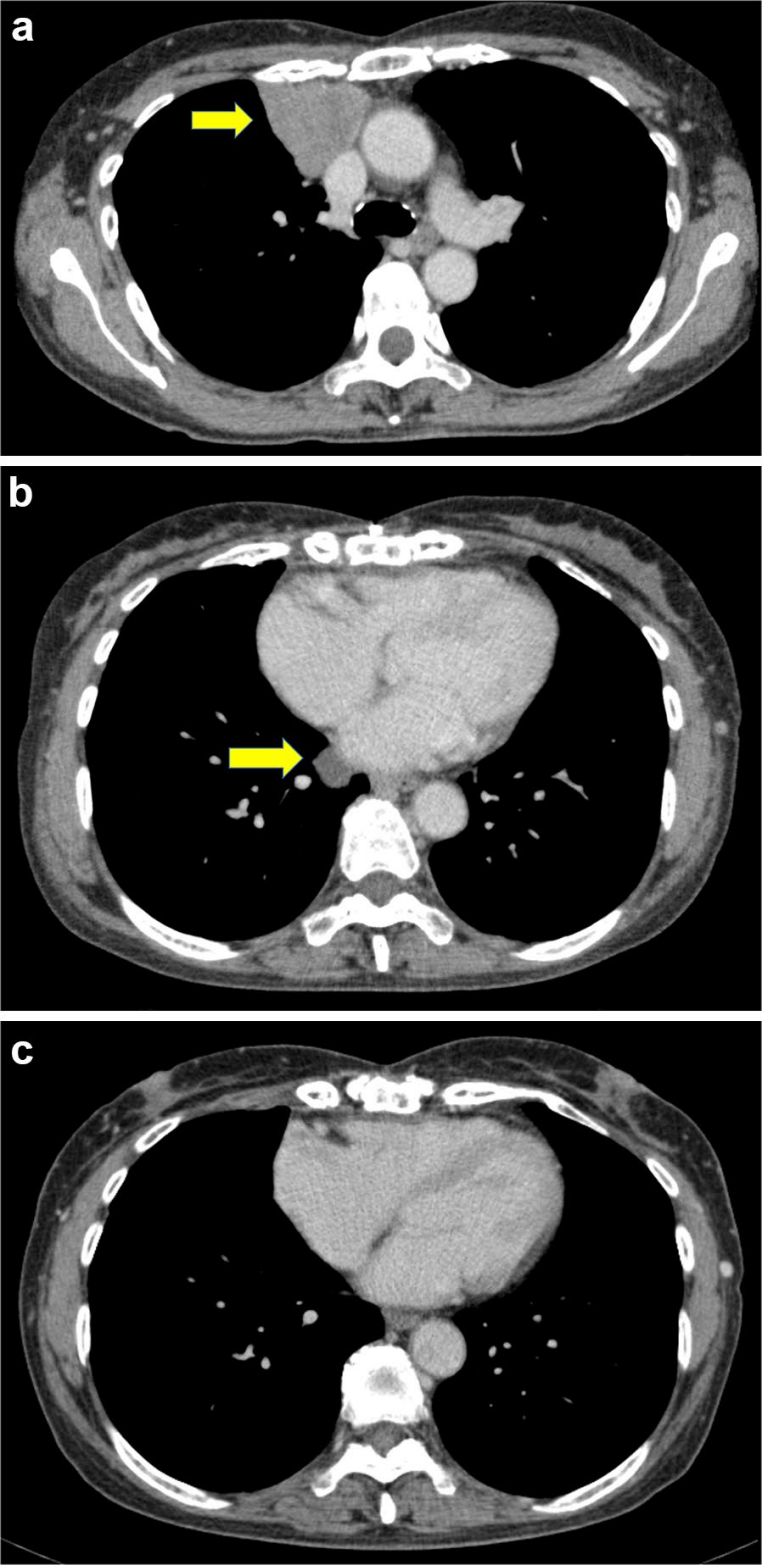
Computed tomography (CT) findings. **a** Local recurrence of thymoma at the first relapse. **b** A pleural dissemination of thymoma at the second relapse. **c** Complete remission following cyclosporine therapy

Ten years later, local recurrent thymoma was identified using computed tomography (CT) (Fig. 1a). The patients received two 3-day courses of methyl prednisolone pulses (1000 mg/day) before surgery, and the size of the tumor decreased following the treatment. She subsequently underwent a complete resection of the recurrent thymoma. The histopathological diagnosis of the recurrent disease was the same as the initial diagnosis. Similar to the initial treatment, she did not receive adjuvant therapy for the recurrent disease.

Three years later, at the age of 63, she began experiencing chronic fatigue and shortness of breath. A blood test revealed that the patient had severe anemia and reticulocytopenia (hemoglobin: 4.6 g/dl, reticulocytes: 1000/μl), and a bone marrow analysis revealed severe erythrocytopenia in the absence of abnormal findings of myeloid cells and megakaryocytes. Based on these findings, the patient was diagnosed with PRCA. A CT scan revealed multiple pleural tumors and swelling of the right hilum lymph node (Fig. 1b), indicating the presence of recurrent thymoma. She was scheduled to receive treatment for PRCA before treatment for thymoma.

The patient began receiving orally-administered cyclosporine in December 2011. The initial dosage of cyclosporine was 5 mg/kg/day, which is the standard initial dosage for PRCA treatment [9], and it was adjusted to maintain serum trough levels of 100–150 ng/ml, which is the lower limit range of normal concentration considering the patient’s age and its renal toxicity. No cyclosporine-associated side effects were observed over the course of therapy. Three weeks after the initiation of cyclosporine therapy, the patient’s hemoglobin levels and reticulocyte count strongly increased, and no blood transfusions were required. Hemoglobin levels reached the normal range in February 2012. In May 2012, CT findings revealed that all of the thymoma lesions had decreased in size, despite the fact that the patient had only been treated with cyclosporine. She continued receiving cyclosporine therapy and was not treated with chemotherapy or radiation treatment. In March 2013, a CT scan demonstrated that the patient was in CR (Fig. 1c). There was an attempt to reduce the cyclosporine dosage in January 2014; however, PRCA relapsed in November 2015 even though the thymoma was in CR. The cyclosporine dose was immediately increased, and the anemia rapidly resolved. Since the PRCA recurrence, the patient has continued receiving cyclosporine therapy (150 mg/day), and both PRCA and thymoma have remained in CR. Now, she has maintained CR of thymoma for more than 3 years without any side effects.

## Discussion and conclusion

Here, we provide the first report of a case of advanced thymoma with PRCA that was cured using only cyclosporine. The findings of this report suggest that cyclosporine has curative potential for the treatment of advanced thymoma.

The therapeutic effect of cyclosporine in thymoma-associated PRCA has previously been demonstrated [7]. Consistent with this finding, PRCA quickly resolved after cyclosporine administration in this case. However, the optimal duration of cyclosporine treatment for thymoma-associated PRCA remains unclear. In the present case, PRCA relapsed after the dose of cyclosporine was reduced, even after 2 years of CR. Sawada et al. reported that cyclosporine discontinuation was significantly associated with relapse in patients with idiopathic PRCA [9]. Therefore, effectively managing thymoma-associated PRCA appears to require the continuation of cyclosporine therapy even after thymoma is completely controlled.

The most noteworthy finding in this report is that cyclosporine induced CR of recurrent thymoma. Cyclosporine inhibits calcineurin, a molecule that plays a key role in suppressing T cells via the T cell receptor signaling pathway. Both cyclosporine and prednisolone are immunosuppressive agents commonly used to treat many types of autoimmune diseases. Although they are not typically used in cancer treatment, some thymoma patients have been reported to respond well to prednisolone treatment [10, 11]. Only a few case reports also indicate the efficacy of other immunosuppressive agents against thymoma. Mochizuki et al. reported that cyclosporine monotherapy alleviated PRCA and reduced the tumor size in patients with advanced B2 thymoma with PRCA [12]. Miura et al. reported that combination therapy with prednisolone and the calcineurin inhibitor tacrolimus reduced tumor size in a patient with invasive thymoma with MG [13]. These findings indicate that some thymoma patients can respond to immunosuppressive therapy administered without cytotoxic agents. Interestingly, the patient in the present report responded to both prednisolone and cyclosporine at different time points. One possibility is that the initial prednisolone treatment influenced the response to subsequent cyclosporine therapy. However, we assume that the responses were independent because the patient received cyclosporine over a period of 2 years after prednisolone treatment following total thymectomy. An independent response to each drug may indicate that prednisolone-responsive thymoma is also responsive to cyclosporine.

The mechanism by which these immunosuppressive agents target thymoma cells remains unclear. However, it is interesting that the benefits of these agents were observed in patients with similar histopathological features. Most patients were diagnosed with WHO histologic classification type B, which is characterized by the coexistence of tumor cells and T lymphocytes [14]. The T lymphocytes in this context are considered non-neoplastic; however, it remains possible that interactions between the tumor cells and lymphocytes promote tumor growth or maintain tumor homeostasis. In that case, cyclosporine or prednisolone may effectively treat type B thymoma by targeting tumor-associated T lymphocytes. Additional insights into this mechanism merit further investigation.

One limitation of the present study is that we could not confirm the pathology of the pleural lesions at the time of recurrence. However, we strongly suspected that the thymoma had disseminated into the thoracic cavity based on the patient’s clinical course and CT findings. The presentation of PRCA further suggested that these lesions represented recurrent thymoma. Although PRCA is a common thymoma-associated complication, it is rarely associated with other types of solid tumors [15].

This is the first report describing the remarkable effect of cyclosporine in a patient with advanced thymoma. As most thymoma patients can be cured by thymectomy, the prevalence and clinical features of cyclosporine-sensitive thymoma are yet to be evaluated. However, these issues should be addressed in future clinical trials. We are optimistic that cyclosporine represents a novel, effective, safe, low-cost therapy for patients with thymoma.

## Abbreviations

CT: computed tomography
CR: complete remission
MG: myasthenia gravis
PRCA: pure red cell aplasia
